# Aggregation of Cricket Activity in Response to Resource Addition Increases Local Diversity

**DOI:** 10.1371/journal.pone.0139669

**Published:** 2015-10-05

**Authors:** Neucir Szinwelski, Cassiano Sousa Rosa, Ricardo Ribeiro de Castro Solar, Carlos Frankl Sperber

**Affiliations:** 1 Programa de Pós-Graduação em Conservação e Manejo de Recursos Naturais, Universidade Estadual do Oeste do Paraná, Cascavel, Paraná, Brazil; 2 Universidade Federal do Triângulo Mineiro, Iturama, Minas Gerais, Brazil; 3 Programa de Pós-Graduação em Ecologia, Universidade Federal de Viçosa, Viçosa, Minas Gerais, Brazil; 4 Departamento de Biologia Geral, Universidade Federal de Viçosa, Viçosa, Minas Gerais, Brazil; Muséum national d’Histoire naturelle, FRANCE

## Abstract

Crickets are often found feeding on fallen fruits among forest litter. Fruits and other sugar-rich resources are not homogeneously distributed, nor are they always available. We therefore expect that crickets dwelling in forest litter have a limited supply of sugar-rich resource, and will perceive this and displace towards resource-supplemented sites. Here we evaluate how sugar availability affects cricket species richness and abundance in old-growth Atlantic forest by spraying sugarcane syrup on leaf litter, simulating increasing availability, and collecting crickets via pitfall trapping. We found an asymptotic positive association between resource addition and species richness, and an interaction between resource addition and species identity on cricket abundance, which indicates differential effects of resource addition among cricket species. Our results indicate that 12 of the 13 cricket species present in forest litter are maintained at low densities by resource scarcity; this highlights sugar-rich resource as a short-term driver of litter cricket community structure in tropical forests. When resource was experimentally increased, species richness increased due to behavioral displacement. We present evidence that the density of many species is limited by resource scarcity and, when resources are added, behavioral displacement promotes increased species packing and alters species composition. Further, our findings have technical applicability for increasing sampling efficiency of local cricket diversity in studies aiming to estimate species richness, but with no regard to local environmental drivers or species-abundance characteristics.

## Introduction

Ecological theory proposes several alternative mechanisms that may drive species diversity. It has become increasingly clear that diversity drivers span multiple spatial and temporal scales [1, 2]. On a regional scale, diversity can be determined by dispersion and colonization events [1, 3], obscuring local determination [4]. On a local scale, ecological interactions may limit diversity via competition for enemy-free space [5] or competition for limited resources [6].

Resource availability is an important driver that can shape the distribution of organisms and ecosystem processes [7, 8]. The diversity response to resource availability can be represented by a hump-shaped curve [9, 10]. Environments with low resource availability can have low diversity, which can be explained by extreme competition [11] or/and dominance [12]. At an intermediate level of resource availability, the environment can support greater diversity due to supporting a greater number of individuals, and hence, more species [13], or by permitting species coexistence [14]. Alternatively, increase in resource quantity may decrease diversity, as providing intra- or inter-specific interactions may cause stress or diminish essential resources [15, 16], and/or by increasing predation pressure [9], rendering some species dominant in the environment. There has been some controversy about the hump-shaped curve, because organism responses are quite variable, and patterns found in nature are often heterogeneous [17, 18].

Crickets (Orthoptera: Grylloidea) are common in forest litter mesofauna, are easily sampled, and experimentally treatable. There is high cricket diversity in the Neotropics, where they occur from ground level up to the tree canopy [19]. Litter crickets have a narrow tolerance range in terms of humidity [20, 21], oviposition sites, and territories [22], however little is known about effects of food resource limitation [23]. Litter crickets are recognized as omnivores, with a primarily herbivorous diet [24]. Despite this broad diet, food can be limiting for crickets because they depend on fruits (sugar), fungi, and animal tissue as diet supplements [25].

In this study, we used a manipulative approach to evaluate if sugar-rich food availability is a limiting resource for litter crickets. We expected that sugar-rich resource addition in the field would (i) increase species richness, by (ii) attracting otherwise sparsely distributed cricket species.

## Materials and Methods

### Study site

**Fig 1:**
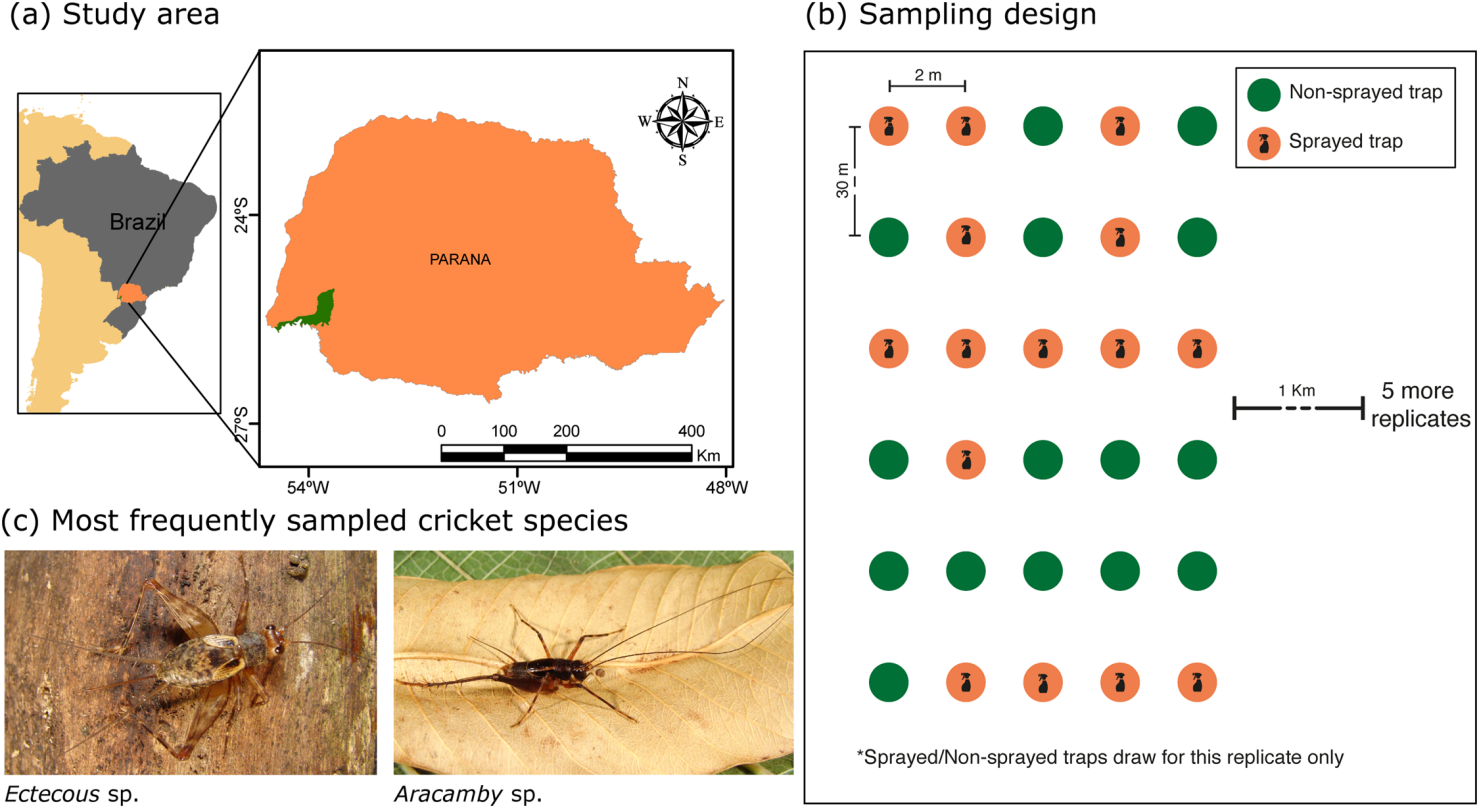
Map of the sampling region (a), sampling experimental design (b) and the most frequent species sampled (c). Maps were created using licensed ArcGIS 10 software. For experimental design, each new replicate we made a new draw to select which traps would be sprayed. Cricket species photos by Pedro G.B.S. Dias.

Sampling was carried out in old-growth Atlantic forest, in the Parque Nacional do Iguaçu (Iguaçu National Park—25°37’35”S—54°27’9”W), Foz do Iguaçu, Paraná state, Brazil, in January 2010. The park area contains 185.262 hectares (Fig 1(a)); it was declared a world natural heritage site by UNESCO in 1986 [26]. Vegetation is composed of tropical semi-deciduous forest and ombrophilous mixed forest, within the Atlantic rainforest biome [27]. The climate in this region can be categorized as humid subtropical mesothermal, with a mean annual temperature of 18—20°C and mean annual rainfall of 1600 mm. The dry and rainy seasons are from April to June and October to January, respectively.

### Experimental design

We created six parallel transects each 180 m in length, 1000 m apart and beginning at a distance of 1000 m from the forest edge. We placed six sets of five pitfall traps (“A” to “E”, each 30 m apart) at each transect. Traps within a set were placed two meters apart from each other and perpendicular to the transect. Traps consisted of polyethylene vials (20 cm diam. and 22 cm depth) filled with 500 ml of ethanol as a killing solution [21]. Traps were left in the field for 48 h. The total sampling effort consisted of 180 traps, aggregated in 36 sets (n = 36).

Treatment consisted of hand-spraying sugarcane syrup (∼ 8 ml) six times on the litter (resource addition spot), up to a 50 cm radius surrounding a pitfall trap. The resource addition was done immediately after the traps were buried into the soil. Within each set of five pitfall traps, the number of resource addition spots ranged from zero to five. The experiment was randomized within each transect (n = 6), where each trap set was equally likely to receive any treatment level (zero to five resource addition spots). Further, within each set, the traps (“A” to “E”) which received the treatment were equally likely to be chosen. Therefore we had 6 treatment levels applied to a range of zero to five pitfall traps within each set (Fig 1(b)). We considered each set of five traps to be a sampling unit (n = 36).

Specimens were identified by the taxonomists Dr. Carina M. Mews and Dr. Pedro G.B.S. Dias, following Desutter’s classification [28, 29]. The national collecting permit was issued by the Instituto Chico Mendes de Conservação da Biodiversidade—ICMBio to NS (SISBio 13543). We obtained all specific permits required to sample in Iguaçu National Park, and the sampled and studied species were neither endangered nor under protection. Specimens were stored in 100% ethanol and deposited in the Laboratory of Orthoptera, affiliated with the Museu Regional de Entomologia da Universidade Federal de Viçosa (UFVB).

### Data analysis

To test if sugar-rich resource addition increased species richness, we adjusted generalized linear models (GLM) with Poisson error distribution, with the number of species per set of five pitfall traps as the response variable (n = 36) and quantity of resource addition as the explanatory variable. We used linear regression to test the response to resource addition quantity, with resource addition quantity as the continuous explanatory variable. To evaluate if the quantity of resource addition affected species richness, we performed a second linear regression excluding the “non addition” treatment level.

We detected evidence of nonlinearity in the species richness response to resource addition. This response was not adequately modeled by polynomial regression, thus we adjusted non-linear regression (*nls* procedure in R) with asymptotic models [30], and evaluated the adequacy of the adjusted models by visual inspection of predicted and observed values. We used Akaike Information Criteria corrected for small sample size (AICc) [31] to choose the best model.

To evaluate whether resource addition worked as bait, attracting crickets to resource-added spots, we tested the influence of resource addition on cricket species richness in two additional, smaller, spatial scales. These spatial scales were hierarchically nested within the sampling units’ spatial scale (among trap sets, with distances > 30 m): within trap sets (10 m) and among traps (2 m). At the smallest spatial scale (*i.e.*, among traps) we evaluated if traps within resource-added spots captured more cricket species and individuals than traps outside resource-added spots. At the next larger spatial scale, we evaluated if cricket captures in traps outside of resource-added spots increased with proximity to resource-added spots. If resource addition spots acted as bait at these spatial scales, then traps within resource-added spots should capture higher species richness than traps outside of the spots (at that spatial scale), and captures in traps outside resource-added spots should increase with proximity to the spots. For both analyses we excluded control transects, were there were no resource-added spots.

To test whether resource addition promoted an increase in the abundance of sparsely distributed species, we adjusted generalized linear mixed models (GLMM), with species identity as one of the explanatory variables, with random intercept and Poisson error distribution [32]. The random effect was pitfall set, nested within transect. The number of individuals in each cricket species per set was the response variable. We performed two-way analysis of variance (ANOVA), with resource addition (2 levels: “no addition” *vs.* “addition”) and species identity as explanatory factors, together with the interaction term. The use of mixed effects models, adjusting resource addition as random effect, enabled avoidance of pseudo-replication [30] by evaluating the effects of resource addition on the abundance of all species in a single analysis. If resource addition affected population size differentially among cricket species by favoring some (*i.e.*, rare species) and reducing others, then the interaction term (species identity *vs.* resource addition) should be significant.

Adjusted models were subjected to residual analyses to evaluate model adequacy and eventual overdispersion. All statistical analyses were done in the R 3.0.3 environment [33]. Raw data of all performed analyses are provided in Supporting Information (S1 Table).

## Results

We collected 1,115 individuals belonging to five families and 13 species. The richest and most abundant family was Phalangopsidae (seven species and 765 individuals), followed by Trigonidiidae (three species and 333 individuals), Mogoplistidae (one species an 14 individuals), Eneopteridae (one species and 2 individuals), and finally Podoscirtidae (one species and 1 individual) (Table 1).

**Table 1:** List of taxa sampled by each resource addition level (zero to five), with their individual and total abundances, sorted by abundance. Family (Ph = Phalangopsidae, Tr = Trigonidiidae, Mo = Mogoplistidae, En = Eneopteridae and Po = Podoscirtidae) and taxon follow Desutter’s classification [28, 29]. Letters A to M refer to the species codes used in Fig 3.

**Code**	**Family**	**Taxon**	**Quantity of resource added per pitfall-set**					
			**0**	**1**	**2**	**3**	**4**	**5**
A	Ph	*Ectecous* sp. Saussure, 1878 [34]	45	88	61	94	145	130
B	Ph	*Aracamby* sp. de Mello, 1992 [35]	17	19	12	19	36	27
C	Tr	*Phoremia zefai* Pereira, Sperber & Lhano, 2011 [36]	3	38	22	36	62	54
D	Tr	*Zuchiella matiottiae* Pereira, Sperber & Lhano, 2011 [36]	-	14	12	15	27	14
E	Tr	*Amanayara* sp. de Mello & Jacomini, 1994 [37]	-	7	1	6	12	10
F	Ph	*Laranda meridionalis* Desutter-Grandcolas, 1994 [38]	-	5	2	13	7	10
G	Ph	*Adelosgryllus rubricephalus* Mesa & Zefa, 2004 [39]	-	4	2	2	11	2
H	Mo	*Ornebius* sp. Guérin-Méneville, 1844 [40]	-	4	4	1	-	5
I	Ph	*Eidmanacris meridionalis* Desutter-Grandcolas, 1995 [41]	-	1	1	1	-	3
J	Ph	*Vanzoliniella* sp. de Mello & Cezar dos Reis, 1994 [42]	-	3	-	-	-	2
K	Ph	*Endecous* sp. Saussure, 1878 [34]	-	1	-	-	-	2
L	En	*Eneoptera surinamensis* (De Geer, 1773) [43]	-	1	1	-	-	-
M	Po	*Brazitrypa paranaensis* (de Mello & Dias, 2010) [44]	-	-	1	-	-	-
**Total**			**65**	**185**	**119**	**187**	**300**	**259**

**Fig 2:**
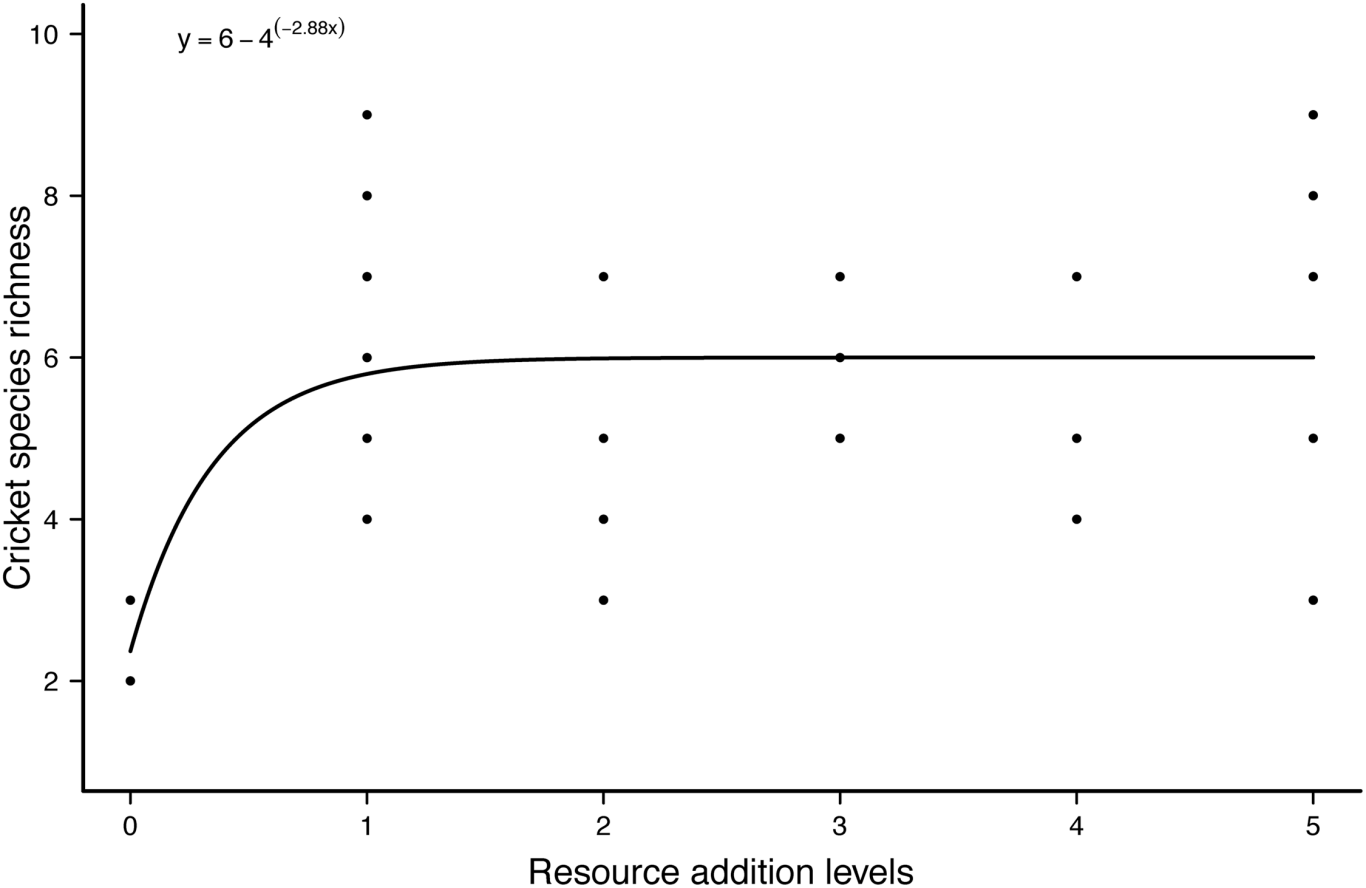
Nonlinear regression describing the asymptotic relationship of the number of cricket species *vs*. resource addition levels.

Linear regression detected an increase in cricket species richness with resource addition quantity (AICc = 150.89; F_1,34_ = 8.48; P = 0.016), but there was no effect of the quantity of resource added once “no addition” samples were excluded (F_1,28_ = 0.095; P = 0.76). Visual inspection suggested a strikingly nonlinear relationship between cricket species richness and resource addition. Nonlinear regression indicated that an asymptotic model was adequate to describe the relationship between cricket species richness and resource addition (AICc = 136.07; F_2,34_ = 7.71; P < 0.001; Fig 2) according to the following equation:

$$\begin{array}{c} y=a-b{\text{e}}^{-cx} \end{array}$$ (1)

We discarded the hypothesis that resource addition worked as bait at spatial scales smaller than our sampling unit. At the smallest spatial scale, traps within resource-added spots did not capture more cricket species than traps outside of resource-added spots (AICc = 536.44; *χ* ^2^ = 3.30; P = 0.069). At the next larger spatial scale, there was no effect of distance to resource-added spots on cricket captures in traps outside of resource-added spots (AICc = 537.63; *χ* ^2^ = 1.42; P = 0.23).

**Fig 3:**
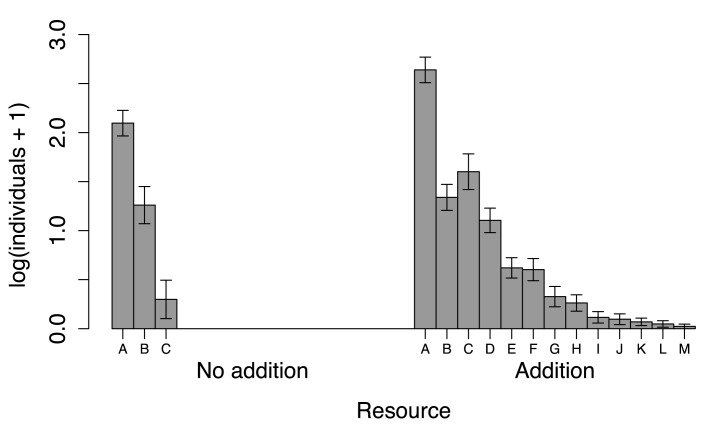
Resource addition effects on cricket abundance. In the figure legend, letters “A” to “M” represent the species captured, as presented in Table 1. In the “no addition” samples three species and 65 individuals were captured, and in the “addition” samples 13 species and 1050 individuals were captured. There was a significant species identity *vs.* resource addition interaction in the “addition” samples. When resource was added, *Phoremia zefai* abundance (C) was three times greater compared to “no addition” samples. With the exception of *Aracamby* sp. (B), all species abundances increased with resource addition levels.

Resource addition affected cricket abundance differentially among species. Using GLMM we detected a significant species identity *vs.* resource addition interaction when evaluated as a two-level factor (*χ* ^2^ = 49.50; P < 0.001, Fig 3). Resource addition did not reduce cricket species abundance. Ten cricket species were exclusively captured in plots with resource addition (*i.e.*, in sites with resource addition, there was a higher abundance of rare species).

The third most abundant species in “no addition” samples, *Phoremia zefai*, was the second most abundant species in samples where resource was added, increasing its abundance by three times. While most species were favored by resource addition, *Aracamby* sp., the second most abundant in the “no addition” samples, were nearly unaffected, as depicted by the overlapping standard error bars (Fig 3).

## Discussion

In this study we provide evidence that increase in resource availability allows packing of species and higher cricket species richness locally, acting as a short-term community driver. The changes promoted by experimental resource addition show that 12 of the 13 cricket species present in forest litter are maintained at low densities by resource scarcity (*i.e.*, food resources are limiting). We propose that this occurs because the crickets perceive sugarcane syrup as a sugar-rich resource, similar to occasionally available fallen fruits, as observed by other authors [45–47]. At such a short time scale (48 h), the local increase in species richness is necessarily due to individual locomotion behavior towards resource-richer areas. The resource pulse increased species richness locally due to behavioral displacement of litter crickets, otherwise sparsely distributed on the forest area, and community structure was altered.

The increase in species richness occurred at a spatial scale of roughly 30 m, with cricket activity increasing near resource-added spots, but without bait-attractant effects within this spatial scale (*i.e.*, the sugarcane syrup did not function as a bait, because traps within resource-added spots did not capture more cricket species than traps outside resource-added spots). Cricket locomotion follows a random walk model [48], increasing the length of stay around a resource; this is a potential mechanism leading to the observed species packing. This could explain why traps within resource-added spots did not capture more cricket species than traps outside of resource-added spots.

Species that occur naturally at low densities have a reduced chance of being sampled, yet experimental increase in resource availability increases species packing. We believe that the underlying mechanism for such a pattern could be associated with the ‘more individuals hypothesis’ [49, 50], which assumes that more resource-rich sites support more individuals, and therefore more species. Here, we provide evidence for a short-term aggregation of individuals, which increases species richness locally. The increase in species richness we observed in response to resource addition is expected, based on the idea that productivity drives diversity [51]. Additionally, we show that cricket species have a large relocating capacity, rapidly increasing their local density and consequently, diversity.

The small spatial scale of our experimental resource addition coupled with observation of cricket responses over a short temporal scale are not sufficient to suggest that resource availability limits population growth (*i.e.*, by limiting births or increasing mortality, [52]). We did not evaluate long-term demographic effects, however we did show that in the short-term [53, 54] forest litter cricket density, and thus species packing, appear to be limited sugar-rich resource availability. Our resource manipulation consisted of a ‘pulse’ feeding resource [55, 56], wherein we instantaneously increased the local availability of sugar-rich resource but did not maintain that resource level in the environment; this momentary pulse of resource input elicited individual displacement behavior. Results from such methods bolster the idea that resource availability acts as a short-term community driver [57]. Sugarcane syrup addition promoted a rapid response, as we measured cricket responses to the enriched environment for only 48 hours. The added resource may have remained available throughout the sampling period, or could have decreased in availability due to resource consumption. Resource runoff was unlikely due to lack of rainfall during the experimental period.

Species composition in the “no addition” samples was treated as a subset nested within species composition in the resource “addition” samples. This, along with the fact that resource addition increased the number of captured individuals, could partially correspond to a shift in Preston’s veil line [13]. When a larger portion of the community was sampled, the probability of capturing rare species increased. Species richness was likely underestimated in the “no addition” samples, because rare species are present at lower densities than those intercepted by the sampling-effort veil line. However, our results diverge from a mere shift in Preston’s veil line [13], because experimentally increasing resource availability led to a change in the species abundance order. For example, there was a three-fold increase in *Phoremia zefai* abundance in the “addition” sites (*i.e.*, where they were the second most abundant) compared to “no addition” sites (*i.e.*, where they were the third most abundant). All but one of the 13 litter cricket species were positively affected by sugarcane addition, which suggests a strong food-resource overlap among these species. When scarcely available, sugar-rich resources limit local species densities, maintaining resource-stressed cricket communities dominated by the three most abundant species: *Ectecous* sp., *Aracamby* sp. (Fig 1(c)) and *Phoremia zefai*.

The results of this study show the displacement capabilities of cricket species, which can rapidly increase their local density and/or diversity in response to attractants. Such results have useful technical implications, as they show that diversity assessment by pitfall traps can be improved by applying sugar-rich resources. Studies aiming to evaluate local species diversity would benefit from this method, as it is an easy and efficient way to increase local species diversity estimation efficiency without increasing the size of the sampling area. However, for studies aimed to evaluate local drivers of diversity, the addition of sugar-rich resource would mask the results because the estimated local diversity would be inflated by active aggregation promoted by researcher interference. For such studies we instead recommend the use of fuel ethanol as a killing solution [21, 58], which improves sampling efficiency without masking local ecological drivers.

Our results highlight sugar-rich resource as a short-term driver of litter cricket community structure in tropical forests. The density of many species appears to be limited by resource scarcity and, when resources are added, behavioral displacement promotes increased species packing and alters species composition. Further, our findings have technical applicability for increasing sampling efficiency in studies of local cricket diversity and species richness, but with no regard to local environmental drivers or species-abundance characteristics.

## Supporting Information

S1 Table This file contains the raw data used in all performed analyses. (XLSX)
